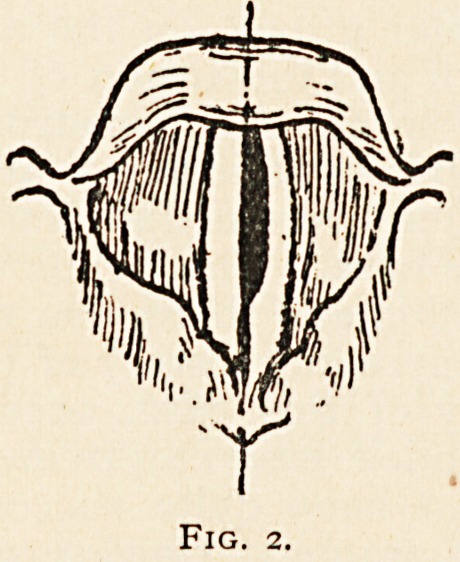# Bristol Medico-Chirurgical Society

**Published:** 1897-06

**Authors:** 


### Bristol Medico-Chirurgical Society.

*March 10th*, 1897.

Dr. A. E. Aust Lawrence, President, in the Chair.

Dr. Barclay Baron showed a lady who consulted him for aphonia, due to multiple papillomatous growths occupying the edges of the vocal cords in their anterior part and the anterior commissure. These were removed with forceps at several sittings, and the site of the growth cauterised with the galvano-cautery, but recurrence occurred with considerable rapidity, and the more the larynx was interfered with the more rapid was the recurrence. Feeling that no endolaryngeal treatment was likely to be of any avail, and being struck with the quite unusual rapidity of the new growth, and in spite of the microscopic examination revealing innocent papillomatous growth, he sent her to Mr. Butlin, who, after removing a piece of growth and examining it microscopically finding it to be of quite innocent structure, agreed with Dr. Baron that thyrotomy was the only means of eradicating the growth. This operation he accordingly performed, removing the warts and cauterising the sites of growth afterwards. The operation was quite successful, except that a web formed which unites the anterior third of the edges of the vocal cords, occupying the former site of the growths. The respiration is good, but the voice is very weak, though not quite aphonic. There are two points of interest in the case: (1) The quite unusual rapidity of recurrence. (2) The danger of web-formation ensuing after thyrotomy for removal of papillomata. We ought to look at the larynx during the progress of healing of the thyrotomy wound, and if agglutination of the vocal cords is taking place, we can then dilate by means of bougies and prevent web-formation.—Dr. P. Watson Williams remarked that this case illustrated some of the unavoidable difficulties and complications which sometimes ensued on the comparatively simple operation of thyrotomy and the great desirability of removal by intralaryngeal methods wherever possible. The fact that these complications were sometimes unavoidable in adults pointed a moral in cases of simple papillomata in children. Unfortunately from their usual site being the anterior third of the larynx, it was often impossible by any means to obtain a good view of the seat of the growth under general anæsthesia, and under such circumstances it was very tempting to gain access to the growth by thyrotomy. But if in adults with a fully developed larynx it was often impossible to avoid injuring the action of the vocal cords, the difficulties and dangers were far greater in young children. Our rule therefore should be to postpone operation in children until they have arrived at the time of life when they can exercise sufficient self-control to permit of intralaryngeal operation in the usual way, and if dyspnœa be present tracheotomy should be performed to allow of such postponement, unless intralaryngeal removal can be accomplished by resort to the laryngoscopic methods of Lambert Lack or Scanes Spicer.

Mr. C. A. Morton showed: (1) A patient with Charcot's disease of the hip-joint. There was enormous effusion of fluid in the joint, and the articular surfaces were separated to a considerable extent. Signs of tabes were well marked. (2) A patient with aneurysm of the third portion of the subclavian artery. For two and a-half months the patient had been kept at absolute rest, direct pressure was applied to the sac, and chloride of calcium administered in large doses. Under this treatment it had decreased in size. (3) An old man with a large warty growth on the tongue. (4) A man with several nodules on the dorsum of the tongue associated with general leucoplakia of the organ. (5) A young man with hypospadias. The urethra opened at the junction of the penis and scrotum. A plastic operation had been performed to lengthen the under surface of the penis as a preliminary to one for the construction of an urethral canal. (6) A child with an enormous (? fatty) growth in the thigh. It was well defined above towards the groin, but seemed to blend with the subcutaneous fat on its lateral aspects.

Dr. Michell Clarke showed: (1) A boiler-maker, aged 58, suffering from athetosis. The movements affect the fingers and toes, are most marked on the left side, and exactly resemble those originally described by Dr. Hammond under this name. The movements dated from four years ago, when the patient suffered from loss of power in the limbs, twitching of the muscles of the left arm, leg, and side of the face, with difficulty in speech, and some mental disturbance. He recovered after a month's illness; but in May, 1896, he had a fresh attack of muscular twitchings on the left side, together with much mental disturbance and delusions. There is now no hemiplegia, nor sensory disturbance, but there is some ataxy of movement of the limbs generally. The visual fields are normal. The movements continue during sleep. An interesting feature in the case is the greatly diminished left knee-jerk. (2) A man, aged 53, with signs of amyotrophic lateral sclerosis. The illness began twelve months ago, with gradually increasing loss of power in the right thigh, first felt by difficulty in going upstairs. Muscular atrophy affects chiefly the right muscular quadriceps extensor femoris, next the right calf muscles. The wasted muscles and also those of the left thigh and calf show altered electrical reactions. There is a well-marked muscular rigidity in the right, less marked in the left lower extremity. Both knee-jerks are exaggerated, the right very greatly. There is no affection of sensation nor of the sphincters, and the upper extremities are normal. With three months' rest in the hospital, electrical treatment and the administration of cod liver oil, arsenic and iron, the affected muscles improved very much. Strychnine was found to increase the rigidity. Since leaving the hospital the paralysis and wasting have made steady progress.—Dr. E. Long Fox called attention to the possibility of convulsion being of use in the diagnosis between athetosis and post-hemiplegic chorea of children; this latter symptom depending usually on sclerosis of part of the cortex.

Mr. W. H. Harsant showed a specimen of cancer of the œsophagus opposite the arch of the aorta, taken from a patient on whom gastrostomy had been performed three months before death. The operation had been perfectly successful, and had given the patient three months of comfortable existence without any of the troublesome after-effects of this operation—such as leakage—which are sometimes described. The patient died suddenly withont any ascertained cause.—Dr. Lacy Firth said that of the special operations to prevent leakage after gastrostomies which had been recently devised, the operation of Albert seemed the best.—Dr. Watson Williams alluded to the long duration of minor symptoms which might eventually culminate in well-marked malignant disease of the œsophagus. He mentioned a case he had recently seen in consultation. The patient first had definite œsophageal obstruction eighteen months before. The obstruction had passed off considerably, so that it had only become a noticeable symptom seven weeks prior to Dr. Williams's examination. Even then the œsophagus readily admitted a No. 16 bougie, and the symptoms did not permit of a definite diagnosis, but within the next seven weeks the patient died with a well-marked growth and considerable glandular enlargement. Dr. Watson

Williams had met with other similar instances.—Mr. Harsant, in reply, said he considered that if the tube accurately fitted the opening, there was no fear of leakage.

Professor Fawcett showed: (1) A skull in which the left lateral sinus had evidently been quite absent, a somewhat uncommon condition. The posterior condyloid foramen was large on that side. (2) A specimen of Meckel's diverticulum, which presented the usual features. (3) A four months' fœtus, which had been expelled along with placenta and membranes entire. (4) Two dissections of the brain, one showing the third ventricle remarkably well, the other displaying the whole length of the nucleus caudatus. Both were hardened and preserved in a 5 per cent. solution of formalin. (5) A dissection of the forearm and hand by Mr. E. M. Pearse. (6) A new model of the deep abdominal viscera, devised by Professor A. Birmingham of the Catholic University of Ireland, and which had been presented to the anatomical museum by Dr. E. Long Fox. Professor Fawcett also described the dissection of the anencephalous monster which had been shown at the meeting in March, 1896 (see *Journal*, 1896, xiv. 192). There were remarkable peculiarities in connection with the nerves (spinal) and the abdominal viscera, an account of which Professor Fawcett hopes to publish later.

Dr. W. H. C. Newnham showed a specimen of ruptured tubal pregnancy.—The President and Dr. Walter Swayne commented on this case (see pp. 152–4).

Mr. C. W. J. Brasher reported "A Case of Division and Ligature of the Vasa Deferentia for Prostatic Hypertrophy," in a man of 87, who had suffered from dysuria for some years, and had had before the operation complete retention of the urine for a fortnight. For some weeks after the operation, the patient suffered from a good deal of mental depression; the catheter was required for six weeks, but it has never been needed since. The prostate was about half its former size two months after the operation.—Mr. Paul Bush related the case of a man over 80 years of age, who came to him some three months ago suffering from an enlarged prostate and commencing cystitis: the patient was then obliged to draw his urine off from forty to fifty times a day, and frequently went to sleep with a catheter in his bladder. Mr. Bush ligatured and divided both vasa; the wounds healed in a week, and within three weeks of the operation the prostate was reduced in size by one-half. When last seen the patient was passing his urine only five or six times in the twenty-four hours, and had discarded the use of the catheter. There were no mental symptoms.—Mr. C. A. Morton drew attention to the fact that in some cases of marked prostatic hypertrophy, in which retention occurred, the power of voluntary micturition may return after a considerable time. He had lately in the hospital an old man of 80, with an enormously hypertrophied prostate, who recovered the power of micturition after four weeks of absolute retention. He left the hospital passing a fair amount of urine at each act of micturition.—Mr. Brasher, in reply, remarked that the return of the power of voluntary micturition was a thing that one often observed; but these cases were probably cases of prostatic hypertrophy complicated by a more or less acute prostatitis, rather than hypertrophy pure and simple.

Mr. Munro Smith narrated "A Case of Dislocation of the Peronei tendons of the Right Leg," in a labourer aged 50. The displacement appears from the history to have come on gradually, and caused sufficient pain to greatly interfere with his work. An attempt was made to keep the tendons in position by an apparatus, but unsuccessfully, and an operation was performed in which the sheath of the tendons was sutured firmly to the fascia. This improved matters, but did not enable the man to work without inconvenience. It was therefore decided to do another operation. The sheath was exposed and constricted in several places with carbolic sutures, and then sewn to the firm fascia behind the groove. A piece of the periosteum was then peeled off the external malleolus, and fastened to the fascia behind the sheath. The man made a good recovery, and after being kept in plaster for four weeks returned to his work, which he can now manage without pain. (This case is more fully reported in the *British Medical Journal* of May 15th.)

---

*April 14th*, 1897.

Dr. A. E. Aust Lawrence, President, in the Chair.

Dr. J. E. Shaw showed a patient, aged 27, suffering from catalepsy, associated with melancholia. The mental condition had developed between Michaelmas and Christmas, soon after which she had become quite cataleptic, remaining in whatever position she was placed, refusing to speak or swallow food. She was always cleanly in her habits. Lately she had been making improvement under massage, strychnine and cannabis indica for medicine, and moral treatment. Though never speaking to strangers, or manifesting physiognomically any mental condition, she is acutely sensitive to what is said to her.

Mr. C. A. Morton showed: (1) A woman, aged 30, from whom he had removed the scapula for a large sarcomatous growth last November. The body of the bone had been broken up and partly destroyed by the enormous growth, but the region of the glenoid cavity and coracoid process was quite free from growth, and so the head and coracoid process were preserved. Special precautions were taken to avoid shock, and this was not marked, nor was hemorrhage serious, the subclavian artery being controlled by digital pressure when the tissues in the neighbourhood of the subscapular artery were divided; the power of movement in the arm is remarkably good. (2) A young man who had suffered from severe attacks of pain in the groin and abdomen associated with an imperfectly descended testicle. The testicle had been transplanted into the scrotum.

Dr. Barclay Baron showed: (1) A case of primary lupus of the nostrils and palate, occurring in a lad 16 years of age, who complained of obstruction and discharge from the left nostril of three months' duration. On examining the nose, it was found that there were masses of pale nodular growth on the anterior part of the septum, the floor of the nostril and the inside of the ala nasi. There was also the same condition in the right nostril, although not so extensive. The uvula was irregularly thickened at its base, and on the naso-pharyngeal surface there were nodules on the uvula and on the palate to the left side of its base. The larynx was quite healthy. The patient had no skin affection and there was no history of such trouble in his family, nor was there any history of phthisis. Dr. Baron, who laid stress on the rarity of this case, occurring as it does without skin affection, recommended free curettement and the application of lactic acid, which had been quite successful in two other cases of an exactly similar character. Care should be taken to remove with the sharp spoon only the diseased tissue, in order to get as little cicatrisation as possible. Recurrence will take place again and again, but eventually cure may be expected. Tonics, such as cod liver oil and arsenic, are useful adjuncts. (2) A case of congenital syphilis in a child 8 years old, who had, as the result of ulceration in the palate and pharynx, extensive web-formation which reached from the palate to below the level of the arytenoid cartilages, and formed a complete membrane except in one place where there was a hole as large as a threepenny-piece. It was thought wise to do nothing for the case, as the only trouble from which the child suffered was regurgitation of fluids into the nose occasionally.—Dr. Watson Williams remarked that cases of primary lupus of the nasal passages were undoubtedly rare. He compared the case under discussion with one he had recently shown, in which a circumscribed sessile, bluish-pink, succulent-looking growth was seen on the right side of the septum strongly resembling a sarcomatous growth, but which on histological examination after removal proved to be a tuberculous growth caseating in the centre. The patient, residing at Bath, did not return for some time, when it was found that the tuberculous deposit had extended to the other nasal passage and then closely resembled the case just seen. The almost universal experience of rhinologists was that although eradication of the disease might be attained by curettement and the application of nitric, chromic, or lactic acid, or the galvano-cautery, there were many cases in which the disease continued to spread despite the most thorough treatment, and his experience accorded with these views.

Dr. Watson Williams exhibited: (1) A case of coarse tremor of the vocal cords. (2) A case of early malignant disease of the epiglottis and right ventricular band. When first sent to him on February 12th, 1897, the glandular infiltration below the angle of the jaw and in the sub-maxillary region was so far advanced as to preclude any hope of surgical removal being attended with any success. (2) A specimen of aortic aneurysm, which was especially interesting as it showed the value of laryngoscopic examination in the diagnosis of intrathoracic tumours. The patient, a man aged 55, came to his out-patient department at the Royal Infirmary on March 22nd last, complaining of hoarseness. True it was that further examination elicited the fact that he had a cough and suffered from dyspnœa, but it was the hoarseness that had attracted his attention. Examination of the larynx revealed abductor paralysis of the left vocal cord and the internal thyro-arytenoid muscle was just beginning to go (Fig. 1), and this, in the absence of any appearance of local disease in a patient past middle life, pointed strongly to aneurysm. The heart's apex was then observed to be displaced downwards and outwards, a soft systolic bruit could be heard over the aortic and pulmonary areas, the aortic second sound being accentuated. Well-

Fig. 1.

Fig. 2.

marked tracheal tugging could be felt and seen, the trachea being dragged down and to the right with each systolic beat of the heart. With the patient in bed, diffuse pulsation of the anterior chest wall was made out. After a few days interval, the internal thyro-arytenoid muscle on the left side became completely paralysed, so that the glottis assumed the appearance shown in fig. 2. The diagnosis then made of aneurysm of the transverse portion of the arch involving the left extremity and causing pressure on the left bronchus, was supported by the physical signs of deficient breath-sounds over the left lung and confirmed at the *post-mortem* examination on April 14th.

Mr. MUNRO SMITH gave the details of an abdominal section he had made on a python for intestinal obstruction. The animal had not eaten for nine months and was very emaciated. On November 20th, 1896, an incision was made and the bowel found very distended, forming a globular tympanitic swelling as large as a football. As no growth or other obstruction could be found, the gut was opened, and a quantity of foul gas evacuated. Then the wound in the bowel was stitched up with catgut and the skin wound with silkworm gut. A few weeks later the wound in the bowel broke down and some fæces passed through it. On January 3rd, the animal ate a guinea pig, and since then has eaten fairly well and passed fæces through the natural cloaca. The wound has healed. It is doubtful what the actual cause of obstruction was.—Dr. A. J. HARRISON said that his experience in somewhat similar cases had not been so satisfactory. He had seen this python frequently since the operation. There could be no doubt about the establishment of the natural passage, because quite recently the animal had passed large masses of fæces through the cloaca.

Dr. W. H. C. NEWNHAM read notes of "A Case of Puerperal Eclampsia." A married woman, aged 22, who was nearly nine months pregnant, had been seized on the afternoon of Dec. 17th, 1896, with convulsions. Chloral and bromide of potassium had been administered in fairly large doses without producing any good effect; the woman was perfectly unconscious and the convulsions were occurring every few minutes. The urine had been found to contain a large quantity of albumen. Chloroform was then given by Dr. Burroughs, who had sent for him, and he began to dilate the cervix; there was no sign of commencing labour. First getting one finger into the cervix, then two, next three and so on, he dilated with his hand sufficiently to get the forceps on to the presenting head and without much difficulty delivered a dead child, the whole operation from the time of beginning dilating with one finger taking only 35 minutes. She had no further convulsion and was quite conscious the next morning. From this time she made an uninterrupted recovery. Her pulse was much too feeble for either venesection or pilocarpin. In every case of repeated convulsions we shall do the best for our patient if we simply empty the uterus.—Dr. WALTER SWAYNE said Dr. Newnham's case appeared to be one in which the treatment adopted was the only one admissible; there is no doubt that when convulsions recur frequently and the pulse and temperature are rising rapidly, immediate delivery is the only treatment. There is considerable divergence of opinion as to the treatment of these cases. The adoption of any routine treatment appears to be a mistake. Prophylactic means will succeed in many cases if undertaken sufficiently early and methodically. The examination of the urine in pregnant women, especially in primiparæ, should be a matter of routine. It has been noted that in patients suffering from albuminuria who are constantly sick throughout pregnancy, eclampsia is rare if not unknown: this seems to show the influence of the digestive functions in adding toxic products to the blood.—The PRESIDENT stated that he considered each case must be treated on its merits, and that this was certainly one for immediate delivery. He also said that if in a given case of puerperal convulsions the use of chloral and chloroform did not check the convulsions then delivery should be undertaken.—Mr. S. H. SWAYNE reported a case he attended in 1863, where finding the patient was plethoric and the urine loaded with albumen, he bled her freely, with the result that consciousness soon returned with no recurrence of convulsions. In about a week natural labour set in, and she was delivered of a healthy living child; the convalescence was normal.

Dr. LACY FIRTH read a paper on "Respiratory Paralysis from Intracranial Pressure." (For this, and the discussion which followed, see pp. 139-45.)

Dr. MICHELL CLARKE read the account of "A Case of Acute Miliary Tuberculosis treated with Antistreptococcic Serum on account of the Presence of S. Pyogenes albus in the Blood." (For this, and the discussion which followed, see pp. 148-51.)

---

*May 12th*, 1897.

Dr. A. J. HARRISON in the Chair.

Mr. JOHN EWENS showed a case of congenital deficiency of both fibulæ and fourth and fifth toes of each foot, with extreme talipes calcaneo-valgus, in a boy nineteen months. The patient, the subject at birth of a breech presentation, was brought to the Children's Hospital when six weeks old. The right foot was then operated on; the left five months later. The tendons of the tibialis anticus, ext. long. pollicis and digitorum, and peronei were cut. It was then found necessary to divide the tendo Achillis, which, drawn out of its normal position, acted as an evertor of the foot. Plaster-of-Paris, and subsequently poroplastic splints were used to maintain position. A light steel apparatus has been used for some months, enabling the child to walk. The comparative rarity of this form of talipes was commented on, and quotations from writers on orthopædic surgery, and papers in the Pathological Society *Transactions*, were adduced. Stress was laid on the depression, or dimple, always found on the most prominent point of the tibial curve. The exact cause is not clear, but it is probably due, according to Mr. Tubby and others, to intra-uterine compound fracture of the tibia. It has, however, been attributed by Nélaton to adhesion of the contracted amnion to the skin of the prominent point where naturally the uterine pressure is greatest, but this does not seem a very satisfactory explanation. Cases of partial deficiency of the fibula were referred to. In all cases of talipes calcaneo-valgus there is a deficiency of two or three of the outer toes, due probably to pressure of the uterine walls preventing development, and there is often irregularity of the tarsal bones.

Mr. C. A. MORTON showed a man aged 40 from whom he had excised the upper three inches of the tibia, containing a myeloid sarcoma, and a corresponding length of the fibula, and screwed the shaft of the tibia to the lower end of the femur, from which he had removed a third of an inch. No vessel or nerve of importance was injured, not even the anterior tibial artery as it passed between the bones. There was four inches of shortening, but with a high-soled boot the patient was able to walk fairly well, three and a half months after operation. Mr. Morton referred to the fact that myeloid sarcomata were often removed together with the portion of the bone in which they grew, when the bone affected was not the main bone of the limb, as in the lower end of the radius or the fibula; but so far as he knew no portion of either the tibia, femur, or humerus had been previously removed for myeloid sarcoma, and the portions of bone above and below fixed together as in this case, but the limb had always been amputated. It was not the fear of recurrence which had deterred surgeons from such conservative surgery, as it was a very well-established practice to excise the portion of bone containing the myeloid sarcoma, rather than to amputate, where the bone affected was not the main support of the limb, but it was rather the fear that removal of a large portion of such bones as the femur or tibia would render the limb useless. This case proved that the fear was groundless, and that amputation need not be done. The portion of bone removed, containing the sarcoma, and a microscopic section of it, were shown.—Mr. Ewens hoped that Mr. Morton would be able to report favourably on the case at the end of a year.—Dr. James Swain wished to add his testimony to the method of securing the bones. Steel pins had been used at the Bristol Royal Infirmary for some years in cases of excision of the knee, and there was no method which gave greater fixation, saved the patient from more pain, or was more likely to be followed by osseous union.—Mr. Morton, in reply, said that amongst sarcomata of bone, myeloid sarcoma occupied a position which was clinically, as well as histologically, totally different from other forms. It did not tend, as a rule, to recur after thorough removal, and so much was this the case, that Bland Sutton had in a paper in the *Edinburgh Medical Journal* for February proposed that they should no longer be called sarcomata, but should be separated from this class and henceforward named myelomata. Mr. Morton thought the patient he had shown was not likely to get any recurrence.

Dr. W. J. Fyffe read a paper on "Fifteen Years' Experience of Infectious Diseases in a Public School." (This will be printed in a future number.)

Dr. J. E. Shaw gave the record of "A Case of Hæmophilia with Joint-lesions." (This will be printed in a future number.)

Dr. G. Parker showed a specimen of hæmato-porphyrin and its spectra from the urine of a patient suffering from lead poisoning. He mentioned that this substance, which was the colouring matter of blood without the iron, though common enough in very minute quantities even in healthy urine, was very rarely found so abundantly as to colour the water a deep port wine colour, as in this instance. It had been observed in various diseases, such as rheumatism, phthisis, and pericarditis, and more particularly after taking sulphonal and trional. In the cases following the use of drugs fatal coma had sometimes been observed, though in others there were no bad symptoms. Hence the practical importance of recognising its excretion. Very little was known as to the way in which it was formed, but probably in some persons sulphonal acted as a corrosive poison. The only treatment which seemed to arrest the process was the administration of large doses of alkalies, though arsenic had also been suggested. This patient had suffered from a second and very rapid attack of lead poisoning just after influenza, with colic and wrist-drop in three days. About the fourteenth day hæmato-porphyrin was noticed, and when the excretion was at its height, severe abdominal pain was felt a little below the end of the spleen. Painful micturition with slight retention came on, but lasted a very short time, and the water became of a dark red colour, acid, without any blood corpuscles, albumen, or reaction for iron or blood. She was then taking iodide of potassium and sulphate of magnesium. Later on a second attack came on during another course of iodide. Lead was found by Mr. F. Stoddart in the urine, and the distribution of the paralysis was that of typical lead poisoning. When the first excretion of the pigment occurred heart failure was very marked, and the patient's state was for a time critical; but a satisfactory recovery was made, and the muscles soon regained some degree of power. The acid and alkaline spectra were shown, as well as the urine which remains undecomposed for a long period. The dark band between D and E in the acid spectrum was strongly marked and was demonstrated.

J. PAUL BUSH, *Hon. Sec.*

---

## Figures and Tables

**Fig. 1. f1:**
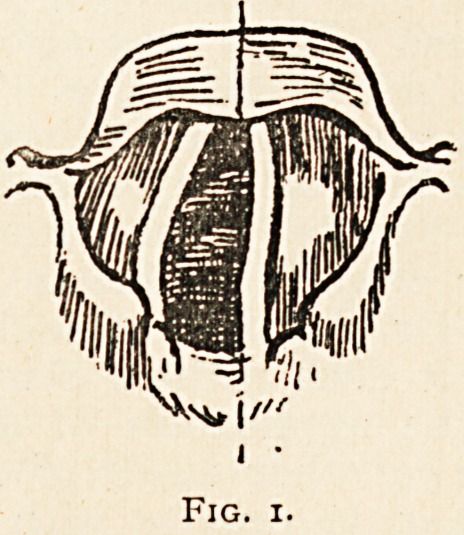


**Fig. 2. f2:**